# Post-kala-azar dermal leishmaniasis and leprosy: case report and literature review

**DOI:** 10.1186/s12879-015-1260-x

**Published:** 2015-11-23

**Authors:** Maria Angela Bianconcini Trindade, Lana Luiza da Cruz Silva, Lucia Maria Almeida Braz, Valdir Sabbaga Amato, Bernard Naafs, Mirian Nacagami Sotto

**Affiliations:** Laboratório de Investigação Médica (LIM-56), Imunodermatologia, Hospital das Clínicas da Universidade de São Paulo, Dr Enéas Carvalho Aguiar 470, 3 andar, prédio 2 Instituto de Medicina Tropical, São Paulo, 05403900 Brazil; Departamento de Dermatologia, Faculdade de Medicina da Universidade de São Paulo, São Paulo, Brazil; Laboratorio de Parasitologia, Instituto de Medicina Tropical, Universidade de São Paulo, São Paulo, Brazil; Departamento de Doenças Infecciosas, Faculdade de Medicina da Universidade de São Paulo, São Paulo, Brazil; Stichting Global Dermatology, Munnekeburen, The Netherlands; Departamento de Patologia e Dermatologia, Faculdade de Medicina da Universidade de São Paulo, São Paulo, Brazil; Rua Cristiano Viana 450, 163, Jardim Paulista, São Paulo, SP CEP: 05411 000 Brazil; Posgraduação Instituto de Saúde, Secretaria de Estado da Saúde de São Paulo, São Paulo, Brazil

**Keywords:** Post-kala-azar dermal leishmaniasis, Visceral leishmaniasis, Leprosy, Leprosy reactions

## Abstract

**Background:**

Post-kala-azar dermal leishmaniasis (PKDL) is a dermal complication of visceral leishmaniasis (VL), which may occur after or during treatment. It has been frequently reported from India and the Sudan, but its occurrence in South America has been rarely reported. It may mimic leprosy and its differentiation may be difficult, since both diseases may show hypo-pigmented macular lesions as clinical presentation and neural involvement in histopathological investigations. The co-infection of leprosy and VL has been reported in countries where both diseases are endemic. The authors report a co-infection case of leprosy and VL, which evolved into PKDL and discuss the clinical and the pathological aspects in the patient and review the literature on this disease.

**Case presentation:**

We report an unusual case of a 53-year-old female patient from Alagoas, Brazil. She presented with leprosy and a necrotizing erythema nodosum, a type II leprosy reaction, about 3 month after finishing the treatment (MDT-MB) for leprosy. She was hospitalized and VL was diagnosed at that time and she was successfully treated with liposomal amphotericin B. After 6 months, she developed a few hypo-pigmented papules on her forehead. A granulomatous inflammatory infiltrate throughout the dermis was observed at histopathological examination of the skin biopsy. It consisted of epithelioid histiocytes, lymphocytes and plasma cells with the presence of amastigotes of *Leishmania* in macrophages (Leishman’s bodies). The diagnosis of post-kala-azar dermal leishmaniasis was established because at this time there was no hepatosplenomegaly and the bone marrow did not show *Leishmania* parasites thus excluding VL. About 2 years after the treatment of PKDL with liposomal amphotericin B the patient is still without PKDL lesions.

**Conclusion:**

Post-kala-azar dermal leishmaniasis is a rare dermal complication of VL that mimics leprosy and should be considered particularly in countries where both diseases are endemic. A co-infection must be seriously considered, especially in patients who are non-responsive to treatment or develop persistent leprosy reactions as those encountered in the patient reported here.

## Background

Post-kala-azar dermal leishmaniasis (PKDL) is a rare skin disease, which may occur after treatment of visceral leishmaniasis (VL) and is encountered mainly in India and in the Sudan, where it was also reported to occur during active VL. It has rarely been reported in the South American literature. It is associated with an inadequate immune response to *Leishmania* and a possible genetic predisposition. It may mimic leprosy clinically with hypo-pigmented macular lesions usually on the face. Histopathological differentiation may be very difficult because both diseases, particularly in the Sudan, may show neural involvement. Co-infection has been reported in countries where both leprosy and VL are endemic and should be considered when there is a poor response to treatment or when persistent leprosy reactions occur [1].

## Case presentation

The patient was a 53-year-old woman who had migrated to São Paulo city, Brazil from Arapiraca, Alagoas in the Northeast of Brazil. She visited the dermatology outpatient department of the University of São Paulo Medical School Hospital (HCFMUSP) with complaints of fever and skin lesions that had persisted for over a year and had worsened during the last 3 months. She mentioned that she had been treated for leprosy (MDT-MB) for 14 months, which had ended 3 months earlier. She had used prednisone intermittently for over a year during outbreaks of reactions.

**Fig. 1:**
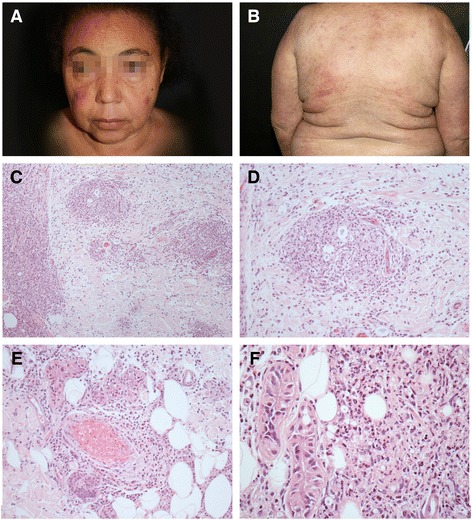
Erythema nodosum leprosum (ENL). **a** and **b** Clinical aspects of ENL episode. **c** to **f** Biopsy from skin lesion displaying nodular macrophage infiltrate (**c** and **d**), vascular thrombosis (**e**) and foci of neutrophils exudation (**f**)

Dermatological examination showed a Cushingoid face, widespread erythematous and edematous, some painful plaques and nodules some of which with central necrosis. This led us to establish the diagnosis of sub-polar lepromatous leprosy (LLsp) with a necrotizing erythema nodosum leprosum (ENL) reaction (type-2 leprosy reaction) (Fig. 1).

She was many times hospitalized during the 3 years after MDT-MB and once in a critical condition with fever, tachycardia and hypotension. Prednisone 40 mg/day and thalidomide 300 mg/day were prescribed at the intensive care unit for treating the reaction. During hospitalization, the patient developed a septic shock. The skin was considered the most likely focus and she was treated with oxacillin.

After 6 days, despite general clinical improvement, the skin lesions worsened, but no other focus of infection was found. Histopathological examinations of the skin, one from a biopsy of the right cavum plantaris and one from a biopsy of the left arm were conducted. Both of them showed multi-bacillary leprosy with a regressive response and fragmented acid-fast bacilli (2+/6+).

After a few days, the patient had new fever attacks, swelling of the abdomen, generalized edema and worsening of the necrotizing ENL. She had elevated levels of liver enzymes, pancytopenia with progressive hypotension, hepatic and hematologic failure. Empirical antibiotic therapy with vancomycin and imipenem indicated by the infectious disease department was started. Liposomal amphotericin B was also introduced to cover the possibility of fungemia or candidemia (the patient had an esophageal candidiasis diagnosed by endoscopy, supposedly due to the long-term corticosteroid therapy).

**Fig. 2:**
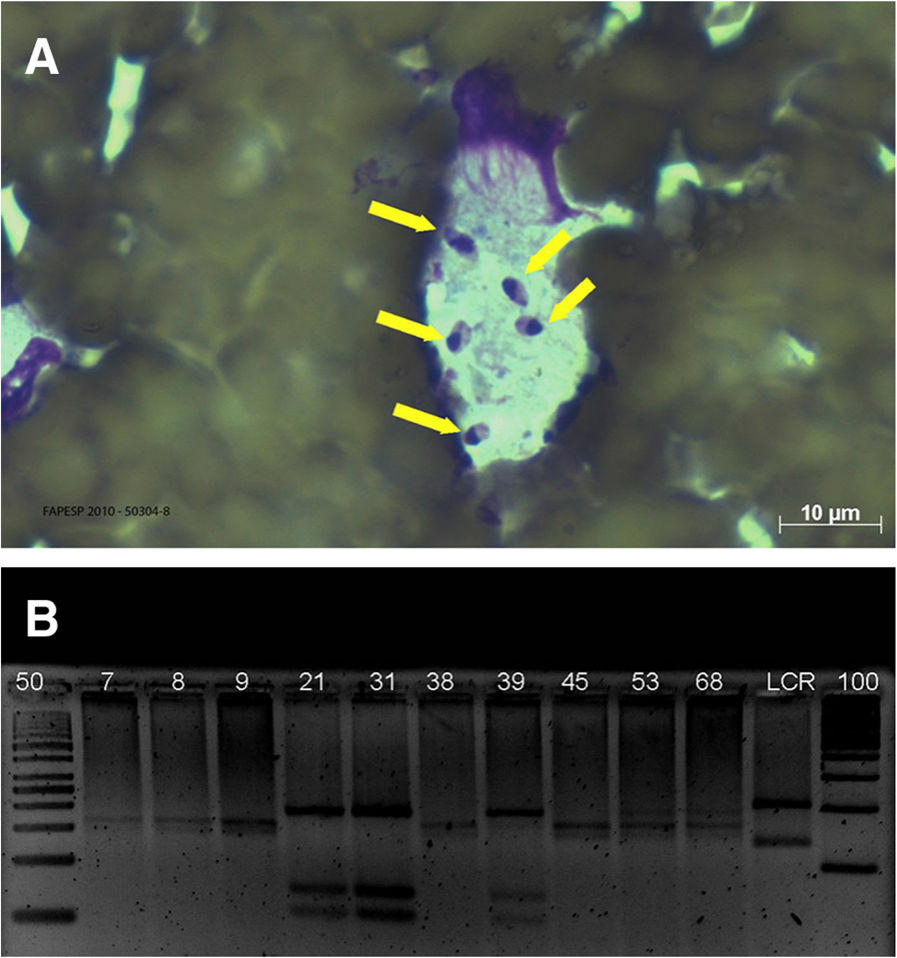
*Leishmania* identification. **a** Smear from patient myelogram displaying amastigotes of *Leishmania* within macrophage cytoplasm (*arrows*). **b** Identification of *L infantum* (184, 72 and 55 bp) in clinical material from a patient (sample 31) using ITS1-PCR-RFLP

Three days later, a new investigation at the emergency department showed hepatosplenomegaly and ascites. VL was diagnosed based on the parasitology of the bone marrow (Fig. 2a), the gold standard for diagnosis of VL and on blood examinations, which showed numerous intracellular and extracellular *Leishmania* parasites; using rk39 (39 amino acid repeats of a kinesin-like gene found in *L. chagasi*), a rapid immunochromatographic test specific for the *Leishmania donovani* complex [2] and by molecular tests (kDNA-PCR = kinetoplast target DNA for PCR and ITS1-DNA-PCR = Internal Transcribed Spacer 1 target DNA for PCR). In addition, after ITS1-RFLP (PCR amplification of the Internal Transcribed Spacer 1 genes (ITS1) and Restriction Fragment Length Polymorphism) (Fig. 2b), fitted-in with the diagnosis of VL, it was demonstrated definitively that *L. (L.) infantum chagasi* was the etiologic agent, because it presented 184 bp (base pair), 72 and 55 bp fragments [3].

Liposomal amphotericin B (4 mg/kg/day for 5 days) was now continued for the treatment of VL (*kala-azar*). This also led to a significant clinical improvement of her ENL skin lesions. Secondary prophylaxis with amphotericin B (3 mg/kg/day for 21 days each month during 6 months) was introduced by the infectious diseases physicians. During the last dose only the kDNA-PCR with her blood was positive.

**Fig. 3:**
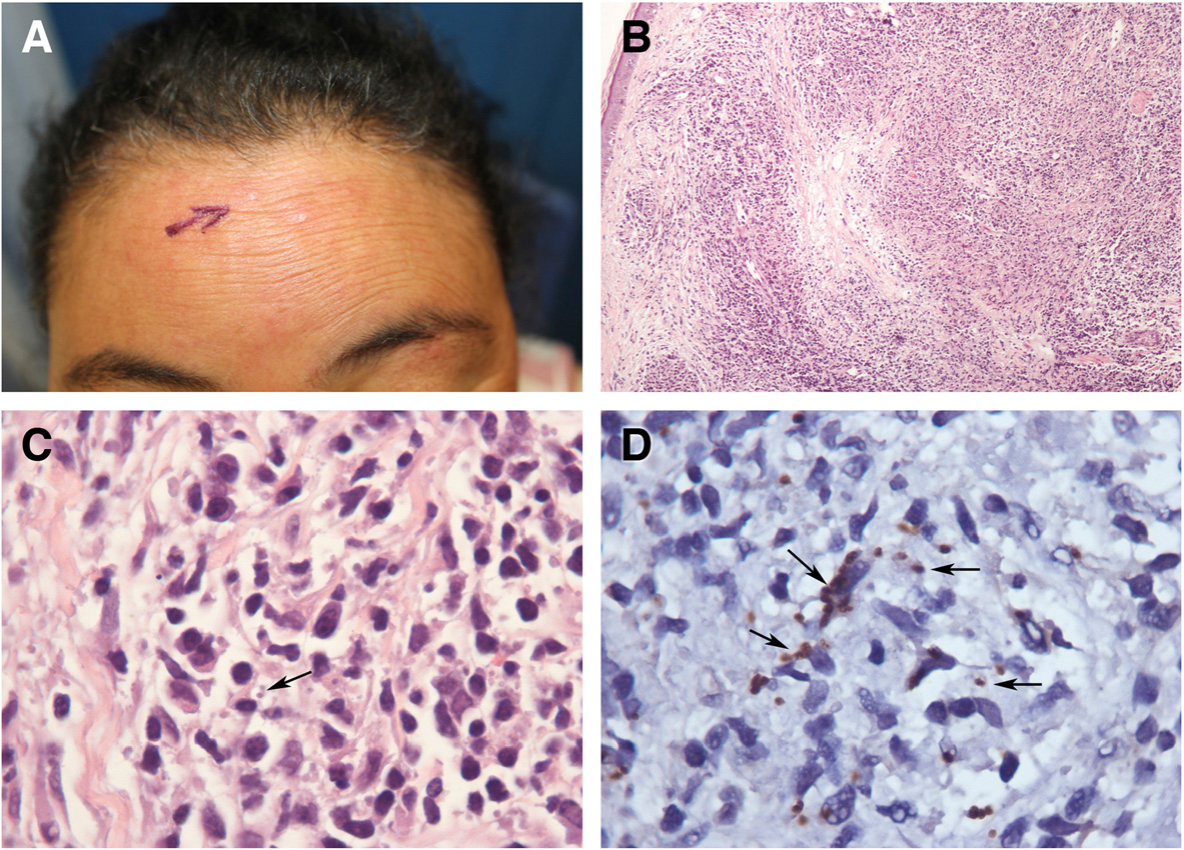
Post-kala-azar dermal leishmaniasis. **a** Hypochromic papule in the forehead. **b** The skin biopsy showed dermal nodular granulomatous infiltrate. **c** Few amastigotes of *Leishmania* were observed within macrophage cytoplasm (*arrow*) intermingled in the lymph plasmacytic infiltrate. **d** The parasites were visible better after staining with anti-*Leishmania* polyclonal antibody

Around 6 months after the treatment for VL and free of necrotizing ENL for 1 year and an occasional nodule of ENL, she developed a few hypochromic papules on her forehead (Fig. 3a). Histopathological examination of a biopsy of such a papular lesion showed a granulomatous inflammatory infiltrate throughout the dermis and superficially in the sub-cutis consisting of epithelioid histiocytes, lymphocytes and many plasma cells (Fig. 3b). *Leishman* bodies were seen within macrophages (Fig. 3c). The presence of *Leishmania* was further confirmed by immunohistochemistry (Fig. 3d) using a peroxidase-anti-peroxidase technique and a polyclonal antibody to *Leishmania* produced in rabbits [4]. At this time, there was no hepatosplenomegaly and the bone marrow did not show *Leishmania* parasites. A revision of all previous skin biopsies with immunohistochemistry for *Leishmania* was performed, but the results were all negative. At that moment, the diagnosis of post-kala-azar dermal leishmaniasis (PKDL) was established and liposomal amphotericin B (3 mg/kg/day for 7 days) was re-introduced resulting in a clinical regression of the skin lesions that was confirmed by histopathological examinations. About 2 years after the treatment of PKDL the patient is still free of PKDL lesions.

## Discussion

Post-kala-azar dermal leishmaniasis is a dermal complication of VL [5], which has been frequently reported from the Indian subcontinent and from the Sudan, where 10–20 % and 50–60 % respectively, of the patients who were treated for VL developed PKDL [6, 7]. In Brazil, only one patient with PKDL was reported and this was associated with AIDS [8].

Visceral leishmaniasis has a worldwide distribution and is considered a public health problem in 88 countries, including Brazil [9]. It is caused by species of the *Leishmania donovani* complex, transmitted by the bite of a female sand fly (*Phlebotomus spp and P. argentipes*) [6, 10]. In Latin America, the main causative agent of VL is *Leishmania (L) infantum chagasi* transmitted by *Lutzomiya longipalpis* [11, 12]. In Brazil, asymptomatic infections and mild forms (low symptomatic) of the disease are more frequent than the classic VL (Kala-azar) [13]. The asymptomatic forms occur especially in children and may heal spontaneously. The determinant factor in spontaneous healing or evolution to fatal classic disease is malnutrition [14].

The symptomatology of kala-azar reactivation in immunosuppressed patients is very variable depending on the type and the duration of immunosuppression (transplantation or autoimmune disease), or the time and the duration of immunosuppression by HIV and others diseases [15]. The clinical forms of VL, particularly in these conditions are very variable. In general, the classic symptoms such as bowel and gastric dysfunctions are not highlight to the diagnosis [16, 17].

In India, the human being is the only known reservoir of VL. This is of epidemiological importance, particularly between epidemic periods of VL. The *Leishmania* parasites survive and propagate intradermally rendering the exposed skin lesions as an easy access area for the sand fly vector to ingest *Leishmania* parasites, get infected and develop the promastigotes in their midgut enabling them to transmit the parasite. The presence of only 0.5 % of PKDL patients during a VL epidemic can potentially succeed in making VL endemic [10]. In other areas such as the Sudan, transmission may be anthroponotic and zoonotic, with rodents and canines as candidate reservoirs [18]. In Brazil, VL is zoonotic with canines as the reservoir host [6].

The causative agent of PKDL in the Sudan and on the Indian subcontinent belongs to the *L. donovani sp.* Of these species *L. (L) infantum chagasi* was also found in the skin lesions of Brazilian patients with VL [19]. In Europe and in South America, PKDL may be considered to be a rare clinical entity among Aids patients with *L. infantum* as the prime causative agent [20]. The same species was also found in the patient reported here.

Post-Kala-azar dermal leishmaniasis may develop during or after treatment of VL [6, 21]. However, some patients have no history of VL and they are easily misdiagnosed as having other skin disorders [18]. In the Sudan, the PKDL cases occur 60 % after VL treatment, 15 % at the same time as VL (called paraKDL), and 10 % even without a history of VL [22]. Indian PKDL appears 6–12 months after the cure of VL [5], whereas in the majority of the Sudanese patients, PKDL occurs within the first 2 months following treatment of VL [23]. It was also reported in HIV/VL co-infected patients receiving HAART [21]. Our patient developed PKDL 6 months after the end of VL treatment, similar to that reported in the Indian patients.

Possible risk factors for developing PKDL include previous VL treatment, its duration and the type of drug used, young age, malnutrition, HIV infection, genetic factors and the parasite strain [24]. Parasite clearance during treatment may have an important influence. Zijlstra et al. [6] reported that patients with a negative tissue aspirate (lymph node or bone marrow) on PCR after VL treatment did not develop PKDL, whereas 36 % of those who were PCR-positive developed PKDL. The patient reported here was PCR-positive in the 4th month of treatment for VL. The presence of a large spleen during VL was also linked to an increased risk of PKDL as were the high serum levels of C-reactive protein as seen in our patient too (data not shown) before treatment for VL, the high level of IL-10 in the peripheral blood and in the normal looking skin during VL [6, 20, 25, 26]. Among all these, incomplete or short treatment of VL seems to be the major risk factor [20]. Our patient had received liposomal amphotericin B (4 mg/kg/day) during 5 days to treat her VL as recommended by the Ministry of Health in Brazil [27] with clinical improvement in few days.

It was argued that after VL treatment there was a subsequent immune activation that could force the parasites to seek refuge within the dermis making this tissue a reservoir for the parasites [10]. Despite the demonstration of PKDL after inadequate therapy for VL in the Sudan, many authors reported PKDL even after adequate treatment with sodium antimony gluconate (SAG), amphotericin B and miltefosine [28, 29]. Today, PKDL is no longer considered to be a specific drug-dependent manifestation [20].

Post-Kala-azar dermal leishmaniasis is clinically characterized by hypo-pigmented macules, erythematous plaques, papular or nodular lesions with *Leishmania* parasites [5, 30]. The lesions generally begin on the face and gradually increase in size. They may also spread to the neck, the trunk and the extremities [10]. Papular or nodular lesions are more common in the Sudanese PKDL, whereas a polymorphic presentation with macules, papules and nodules is more common in the Indian patients [6, 31, 32]. Unusual clinical variants such as papillomatous, verrucous, hypertrophic, xanthomatous, annular and lupoid lesions have also been reported [33, 34]. Mucosa involvement in PKDL is very rare [35]. No constitutional symptoms have been reported [36].

Although clinical forms may differ in different countries, the hypopigmented macules on the face are generally the first lesions to appear in PKDL. Single lesion occurs in up to 5 % and 10 % respectively, of the PKDL cases in Africa and India [30, 37]. Our patient presented with only a few hypopigmented papules on her forehead as the unique manifestation of the disease.

The diagnosis is established after assessing the clinical signs and symptoms [38]. History of VL, living in an endemic area and positive antibody tests are helpful in the diagnosis [20, 38]. According to Zijlstra et al. [6], it is important to observe the type of rash as well as its distribution (in the Sudan, the rash generally begins around the mouth, then spreads to the nose and the cheeks and finally to other parts of the face and the body) and the time relation to VL treatment. However, the ideal diagnostic method is to demonstrate the parasite in smears, culture or PCR [18, 20].

In all clinical types of PKDL, histopathological examination of the skin biopsy shows an epidermis with hyperkeratosis, acanthosis or atrophy and hydropic degeneration of the basal layer. The presence of parasites in biopsies varies with the type of the rash and the duration of the lesions. The biopsy from macular lesions usually consists of sparse inflammatory infiltrate of lymphocytes, histiocytes and a few plasma cells predominantly around the vessels of the superficial vascular plexus. *Leishmania* amastigotes are usually absent in such lesions, but the presence of plasma cells is an important clue in favor of PKDL. Biopsies from the papules and plaques show a moderate to dense lymphocytic inflammatory infiltrate in the mid-dermis, with histiocytes and plasma cells. In nodular lesions, the histopathological examination of the biopsy shows a diffuse dermal inflammatory infiltrate consisting of histiocytes and plasma cells in large numbers. Compact epithelioid granulomas may also be observed [6].

The *Leishmania* amastigotes are intracytoplasmic structures in histiocytes. Amastigotes are observed in 25–50 % of the hematoxylin-eosin-stained biopsies of nodular and plaque lesions of PKDL [39, 40]. In the patient reported here, the papular lesion had *Leishmania* amastigotes, which was also confirmed by immunohistochemistry with an anti-*Leishmania* polyclonal antibody.

Neuritis involving small cutaneous nerves similar to that in leprosy was reported in PKDL. However, peripheral large nerves are not involved in PKDL [6, 36].

Leprosy is the main differential diagnosis in PKDL because of the clinical and histopathological similarities [30], but their differentiation may be very difficult [36, 41, 42]. The small hypopigmented lesions seen in PKDL are very similar to those in borderline and lepromatous leprosy [30, 41]. The patients with leprosy and PKDL usually come from the same geographical location where both diseases are endemic [5]. However, leprosy is associated with hypoesthetic lesions [22]. Arora et al. reported that the center-facial involvement and the sparing of ear lobes in PKDL may be distinguishing features from leprosy [5]. Histologically, PKDL displays epithelioid cell granulomas, similar to tuberculoid leprosy. The parasites may be absent in the macular variant of PKDL making it essential to exclude other diseases [30]. Perineural infiltration in PKDL was reported to cause great difficulty in differentiating PKDL from leprosy [30, 35]. However, the main histological difference between these diseases is that in the macular lesions of leprosy, the inflammatory infiltrate is centered in the neurovascular plexus in the lower dermis. Besides this, in the nodular lesions of lepromatous leprosy, the peripheral limits of the infiltrate are infiltrative, whereas those in nodular PKDL have a fairly sharp margin [30].

Leprosy and leishmaniasis are both spectral diseases and co-infections have been reported. Although it is a rare association, it occurs in countries such Ethiopia and India where both diseases are endemic [42]. The case reported here of co-infection with PKDL is the first in Brazil.

Leishmaniasis and leprosy share a lot of similarities. Both diseases are caused by obligate intracellular organisms. The clinical and pathological expressions depend on the host response, probably due to genetic determination and environmental influences [42, 43]. At the hyperergic pole, the patient shows localized lesions with well-formed granulomas with few or absent organisms, whereas at the anergic pole, the lesions are widespread, there is no epithelioid granulomatous reaction and there are numerous parasites [42].

It was suggested by Bansal et al. that there is a cross-protection between *Mycobacterium* and *Leishmania* infection since both diseases increase macrophage activation. However, the immune deficiency in leprosy is apparently specific for *M. leprae* only and does not dictate the immune response in leishmaniasis. They reported a patient in whom the macular variant of PKDL (‘low-resistance’) coexisted with ‘high-resistance’ borderline tuberculoid leprosy [42]. Our patient presented subpolar lepromatous leprosy with a VL co-infection considered as an anergic pole of both diseases (Th2 response). After developing PKDL, the immunological response changed to the hyperergic pole (Th1 response) of the *Leishmania* infection, when she showed papules with epithelioid granulomas, but she remained at Th2 pole of leprosy indicating that the immune defect was specific for each microorganism [42, 43].

To date, there is no consensus and no large studies on the best available treatment for PKDL. Moreover, the evolution of this disease differs in different geographical regions. Fifty per cent of the Sudanese PKDL is self-limiting and heal spontaneously, whereas all Indian PKDL cases required treatment [6, 20, 33]. In cases of severe lesions or lesions persisting for more than 1 year in the Sudan, treatment with sodium stibogluconate (SSG) 20 mg/kg/day for 1–2 months was instituted [6, 10]. Miltefosine administered orally was an effective and safe treatment for Indian visceral leishmaniasis. It may protect against PKDL because it is given for a much longer period. It may be helpful in regions where the parasites are resistant to the current agents [15, 44].

In cases of SSG-unresponsive treatment, amphotericin B 2 mg/kg/day for 20 days was reported to be effective [6, 10, 20]. Other therapeutic options include miltefosine, ketoconazole and pentamidine [6, 10]. Clinical cure may differ according to the PKDL type. Usually nodules and plaques disappear in 120 days and macular lesions in 200 days [6, 10]. However, parasitological cure may precede the clinical cure, and long treatment regimens need to be carefully monitored [6]. Our patient received liposomal amphotericin B (3 mg/kg/day for 7 days), with clinical healing of the skin PKDL lesions in 3 months.

At this moment the patient is in good health and without skin lesions indicative of PKDL. She has only regressive macules of leprosy. However, at present she received the 10th dose of a newly started MDT-MB treatment because she had the first symptoms of tibial neuritis about 5 year after leprosy treatment. It is likely that the previous treatment for 14 months may have been inadequate.

## Conclusions

Post-kala-azar dermal leishmaniasis is rare and generally occurs after treatment of VL. It shows geographical variation in its clinical presentation and may be multifactorial (genetics of the patient and or parasite, treatment, nutrition and or co-infection). It may mimic leprosy and that must be recognized, especially in countries where these diseases are endemic. Increasing globalization with large number of people travelling should also be seriously considered.

A co-infection may provoke more morbidity and may also lead to more disability. A co-infection should be considered especially in patients not responding to treatment or have a persistent leprosy reaction similar to that described in the patient reported here.

Finally we introduce a serious concern: Visceral leishmaniasis will be able to become an anthropozoonosis in the Americas, like in India (15), because, before and during PKDL, the parasite will be exposed in the skin.

## Consent

Written informed consent was obtained from the patient for publication of this case report and any accompanying images. A copy of the written consent is available for review by the Editor of this journal.

## Abbreviations

Bp: Base pair
ENL: Erythema nodosum leprosum
HAART: Highly active antiretroviral therapy
HIV: Human immundeficiency virus
ITS1-PCR: Internal Transcribed Spacer 1 target DNA for PCR
ITS1-RFLP: PCR amplification of the Internal Transcribed Spacer 1 genes (ITS1) and Restriction Fragment Length Polymorphism
kDNA-PCR: Kinetoplast target DNA for PCR
LLsp: Sub-polar lepromatous leprosy
MDT-MB: Multidrug therapy multibacillary
PCR: Polymerase chain reaction
PKDL: Post-kala-azar dermal leishmaniasis
rk39: 39 amino acid repeats of a kinesin-like gene found in L. *chagasi*
SAG: Sodium antimony gluconate
SSG: Sodium stibogluconate
VL: Visceral leishmaniasis
